# Influence of Cold Wave Diversities on Thermal Stress and Thermal Fatigue Life of Asphalt Mixture

**DOI:** 10.3390/ma17112541

**Published:** 2024-05-24

**Authors:** Haimei, Lili Li, Qinglin Guo, Wenli He, Aosen Xu

**Affiliations:** 1 Faculty of Road and Bridge Engineering, Inner Mongolia Vocational and Technical College of Communications, Chifeng 024005, China; 2 School of Civil Engineering, Hebei University of Engineering, Handan 056038, China

**Keywords:** thermal stress, cold wave, asphalt mixture, thermal fatigue life, stress threshold

## Abstract

Apart from low-temperature cracking, asphalt pavement may also suffer from thermal fatigue cracking. To clarify the impact of cold waves on the thermal fatigue performance of asphalt mixtures, the typical atmospheric temperature characteristics of different regions in China from 2012 to 2019 were analyzed, and the frequency of cold waves in these regions was determined. The viscoelastic parameters of an asphalt mixture are determined through an indirect tensile relaxation test. The thermal stress of the asphalt mixture is simulated and analyzed by using the finite element method. The effect of cold waves on the thermal fatigue life of the asphalt mixture was evaluated. The results show that the frequency of cold waves is different from region to region in China, and the cold waves mainly occurred from October of one year to February of the next year. Northeast China has the most frequency and the largest temperature drop amplitude, followed by North China. The maximum thermal stress increases with the decrease in temperature drop and initial temperature and is unrelated to the duration of cold waves. The thermal stress calculated based on the atmospheric thermal boundary is higher than the value using the road surface temperature. The thermal fatigue lives of asphalt mixtures in North China and Northeast China are very short, while the thermal fatigue life of the mixture in Central China is the longest. To meet the requirement of thermal fatigue damage caused by cold waves during the designed service stage, the recommended threshold for thermal stress is 0.39–0.77 MPa.

## 1. Introduction

Asphalt mixtures are typical thermal-sensitive materials, greatly influenced by temperature variations. Changes in atmospheric temperature induce alteration in the temperature field of the asphalt layer, leading to thermal stresses. Consequently, the impact of thermal stresses on the cracking resistance of pavement cannot be overlooked. Research has identified two main causes for the generation of thermal stresses. Firstly, differences in thermal properties among various layers of road structure result in varying contracted deformation when temperature changes. The road layers are mutually constrained, thus generating thermal stresses in asphalt pavement. Secondly, asphalt pavements are significantly affected by solar radiation and atmospheric convection heat exchange. The combined effects of these factors create a temperature gradient within the pavement structure, thereby generating thermal stress. Fluctuation in the thermal stress can trigger thermal cracks in asphalt pavement, with cracks potentially extending downward until they penetrate the entire structure, thereby affecting road driving comfort. Existing methods for determining thermal stresses in asphalt pavement typically involve numerical simulation, theoretical analyses, and laboratory experiments. Although the use of polymer-modified asphalt can enhance the low-temperature crack resistance of asphalt mixtures [1], due to the complex service environment of asphalt pavement, theoretical methods may fail to analyze the influence of complex temperature history. Consequently, the numerical simulation method is widely employed to analyze thermal stress in asphalt pavement [2,3].

Apeagyei et al. [4] employed the finite element method to analyze thermal stresses in asphalt pavements, revealing a significant correlation between thermal stress and initial temperature as well as cooling rate. Tian et al. [5] conducted thermal stress restrained specimen tests (TSRSTs) to examine the thermal stresses of two types of asphalt mixtures under various initial temperature and cooling rates, thereby refining the temperature–thermal stress relationship. Their analysis indicated that when the initial temperature was above 5 °C, the asphalt mixture exhibited excellent relaxation capabilities, resulting in no thermal stress regardless of the cooling rate. Similarly, Tan [6] analyzed the thermal stress in asphalt pavement above 0 °C under the non-periodic cooling condition, revealing that regardless of the initial temperature and cooling rate, when the temperature drop interval is above 0 °C, thermal stress in asphalt pavement is very small. The accumulated thermal stress is far less than the ultimate tensile strength of the asphalt mixture, thus preventing low-temperature-induced cracking. Fan et al. [7] conducted a virtual TSRST simulation, indicating that the asphalt mixture exhibited good relaxation capability at middle temperature, resulting in minimal cumulative thermal stress. As the temperature gradually decreased below 0 °C, thermal stress in asphalt pavement increased. Pszczola et al. [8] determined the master curve of modulus using bending creep experiments and direct tensile creep experiments. They calculated the thermal stresses of the asphalt mixture using different methods, pointing out that the strain caused by tensile creep at low temperature is smaller, and the thermal stress of the asphalt mixture can be calculated using the results of the direct tensile creep test with good reliability. Yang et al. [9] proposed an equivalent fracture temperature (EFT) corresponding to the critical fracture temperature in the TSRST, and the results showed that the EFT is as accurate as the critical fracture temperature in evaluating the low-temperature cracking performance. Compared with fracture energy and critical fracture temperature, the EFT is more sensitive to the type of mixture and can effectively distinguish the low-temperature performance of different mixtures. Wang et al. [10] studied the low-temperature performance of different mixtures using a low-temperature bending test and TSRST experiment, pointing out that the fracture temperature is the best indicator for describing low-temperature performance and can effectively differentiate asphalt mixtures in high-altitude and cold regions. Arabzadeh et al. [11] developed a setup to measure the thermal fatigue performance of the asphalt mixture under a constant strain cyclic loading pattern, and the results indicated that the asphalt content, aggregate type, and binder type have the strongest influence on the thermal fatigue resistance of the asphalt mixture. Zhou et al. [12] pointed out that at a constant initial temperature, thermal stress in the mixture increased with the cooling rate, a lower cooling rate yielding a smoother thermal stress trend because of the relaxation. For the constant cooling rate, a lower initial temperature resulted in a higher thermal stress upon reaching the same temperature [13]. Sun et al. [14] noted that thermal stresses in various layers of the road increased with the temperature fluctuation range, and the surface layer is the most affected by the daily temperature variation. Xiao et al. [15] studied thermal stresses in asphalt surface layers under the influence of daily temperature, indicating that the thermal stresses in asphalt surface layers exhibited a sinusoidal trend. In the rapidly cooling segment, thermal stress accumulated due to insufficient relaxation ability. Prolonged low-temperature conditioning decreased the stress relaxation capability, accelerating pavement thermal damage.

Meanwhile, Das et al. [16] analyzed the effects of critical fracture energy and the nonlinear thermal shrinkage coefficient on the thermal cracking of the asphalt mixture based on the finite element method, which was used to predict the thermal stress and fracture temperature. The results indicated that the thermal shrinkage coefficient and cooling rate had significant impacts on the fracture temperature of the asphalt mixture. Gu et al. [17] conducted pavement cracking analysis using a numerical method, suggesting that when the strain energy caused by the load exceeds the ultimate strain energy of the asphalt mixture, cracks will occur, thus determining the initial life of top-down cracks. Ling et al. and Onifade et al. [18,19,20] studied the non-load-related failure mode of asphalt pavement, specifically thermal cracking. They utilized finite element simulation to determine the J-integral at the tip of thermal cracks, applying it to the Paris law to predict the crack extension over time and fatigue life. Wang et al. [21] investigated crack propagation behavior in the asphalt mixture, validating the reliability of the finite element method through indirect and direct tensile tests on asphalt mixtures. Hasni et al. [22] and Ling et al. [23] studied the crack propagation law of asphalt pavement under the coupling effect of thermal and traffic loads and analyzed the evolution of top-down cracks in asphalt pavement. Multiple indicators considering the mixture damage were used to depict the crack propagation process of asphalt layers. Das et al. [24] investigated the influence of the fracture energy of different mixtures on thermal cracking through numerical simulation and experiments. They concluded that the thermal shrinkage coefficient, cooling rate, and creep compliance significantly contribute to thermal stress. A nonlinear thermal shrinkage coefficient provided a much better prediction than the linear approach. In 2018, Rahbar-Rastegar et al. [25] employed three methods to analyze the fatigue and crack resistance performance of nine asphalt mixtures. The results indicated that the integration of simulation models is crucial for establishing a reliable connection between lab-measured properties and the cracking behaviors of the asphalt mixture. Additionally, the research demonstrates that fatigue and low-temperature cracking performances exhibit no correlation.

Based on the above studies, it can be observed that thermal stress in asphalt pavement primarily occurs at low temperature (<0 °C). The asphalt mixture has good relaxation capability and will not generate thermal stress when the temperature is above 0 °C. The cooling rate significantly affects thermal stress, and a higher cooling rate results in larger thermal stress. The concept of low-temperature cracking suggests that as long as thermal stress does not exceed the ultimate tensile strength of the asphalt mixture, cracking will not occur [2]. However, Song et al. [26] pointed out that while thermal stress did not exceed the ultimate strength, thermal fatigue damage may occur within the mixture. Lv et al. [27] indicated that the fatigue life of the asphalt mixture is in a power function relationship with the ratio of thermal stress, high thermal stress leads to a shorter fatigue life of the asphalt mixture. Khan et al. [28] confirmed that temperature variations are diverse in different regions, with regions located in higher latitudes experiencing lower temperature. Additionally, cold waves differ across regions, inevitably exerting a significant influence on the thermal stress of the asphalt layer. Therefore, studying the characteristics of cold waves in different regions and analyzing their impacts on thermal stress is helpful for predicting the thermal fatigue life of the asphalt layer.

In light of this, this paper takes some regions of China as an example, conducts statistical analysis on the daily temperature, determines the occurrence frequency of cold waves, performs an indirect tensile relaxation experiment to obtain the viscoelastic parameters of the asphalt mixture, analyzes the variations in thermal stress based on FE simulation, and predicts the thermal fatigue life of the asphalt mixture. This work provides a new perspective for predicting the thermal fatigue life of the asphalt layer. This makes it possible to estimate the thermal fatigue performance of asphalt pavement using cold wave statistical data, developing a method for the design of the thermal fatigue performance of the asphalt layer in different regions.

## 2. Materials and Methods

### 2.1. Materials

**Figure 1:**
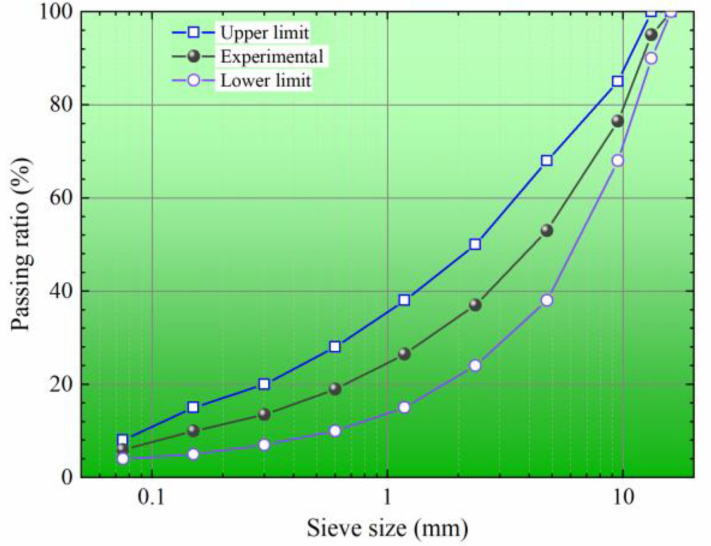
Experimental gradation.

The experiment adopts AH-70# and AH-90# petroleum asphalt from Sinopec Oilu Petrochemical Company (Beijing, China) with penetration grades of 70 and 90 to prepare the mixtures. Its essential characteristics are detailed in Table 1. Basalt aggregates, along with their apparent gravity and moisture uptake ratio, are outlined in Table 2. The experiment adopts the AC-13 gradation recommended by China’s technical specification JTG D50-2017 [29] for the dense mixture depicted in Figure 1. Based on Marshall test outcomes, the optimal asphalt content is determined to be 4.8%, resulting in a mixture apparent gravity of 2.457 g/cm^3^ and an air void of 3.8%.

### 2.2. Indirect Tensile Relaxation Test

**Figure 2:**
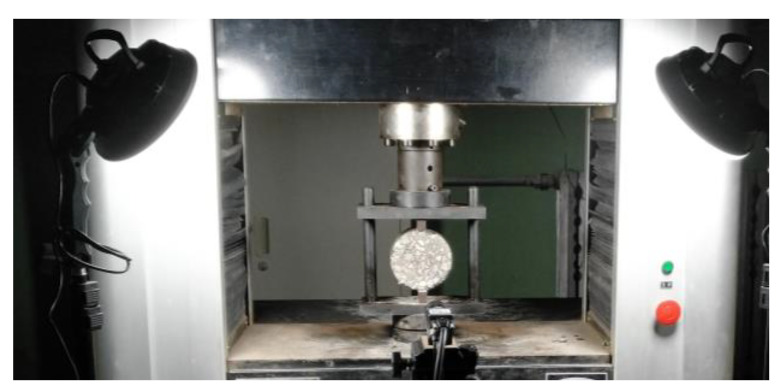
Indirect tensile stress relaxation test.

This work selects the indirect tensile relaxation test to measure the viscoelastic parameters of asphalt mixture [36]. Due to the limitation of loading rate, the initial constant strain cannot be applied instantaneously, and a loading time is required. In the early loading stage, the loading rate of 1 mm/min is adopted to make the indirect tensile strain reach the set value according to the Chinese specification JTG E20-2011 [37]. The specimen dimension is 101.6 × 63.5 mm. In order to ensure that the asphalt mixture is relaxing completely in the linear viscoelastic stage, 30% of the ultimate tensile strain is taken as the set value in this work. The digital image correlation (DIC) is used to measure the strain field of asphalt mixture. The specimen surface is polished to obtain the characteristic of random speckle [38]. The process of indirect tensile relaxation test is shown in Figure 2, and the experiment is conducted at 20 °C due to the limitation of equipment.

The indirect tensile relaxation modulus can be calculated using the following Equations (1) and (2).

(1) $$E=\frac{\sigma \left(t\right)}{\varepsilon_{0}}$$

(2) $$\sigma \left(t\right)=\frac{0.006287P_{T}}{h}$$

where *P*_T_ is the applied load, N; *ε*_0_ is the initial strain value; and *h* represents the height of the specimen, mm.

The constitutive relationship of asphalt mixture can be described using the generalized Maxwell model, and the relaxation behavior can be represented using Prony series. And the relaxation modulus parameters can be fitted using the following formula.

(3) $$E\left(t\right)=E_{\infty }+\sum_{i=1}^{m}E_{i}e^{-\frac{t}{\tau_{i}}}$$

where $E_{\infty }$ is the long-term relaxation modulus. To determine the parameters of Prony series, one should first select the relaxation time $\tau_{i}$, and then nonlinear fitting is used to determine the modulus parameters $E_{i}$. To ensure the fitting accuracy, this paper uses a five-element generalized Maxwell model to fit the curve of relaxation modulus. The viscoelastic parameters are shown in Table 3. It should be noted that AC-13-70 represents asphalt mixtures prepared using AH-70# asphalt.

(4) $$\mathrm{lg}\alpha_{T}=\frac{C_{1}\left(T-T_{0}\right)}{C_{2}+\left(T-T_{0}\right)}$$

The relaxation modulus at any temperature can be determined by introducing a temperature shift factor, and the calculation method of the shift factor is shown in Equation (4). According to the research results of Sun et al. [39], for 70# and 90# asphalt mixtures, it is recommended to use the parameters as shown in Table 4.

### 2.3. Statistical Analysis of Cold Waves

Cold waves are weather events that cover a wide area and mostly occur in autumn and winter, causing a sudden drop in temperature. According to the Chinese national standard: Cold Wave Grade (GB/T 21987-2017) [40], cold waves include three levels: cold wave, severe cold wave, and extreme cold wave based on the daily minimum temperature and the temperature drop range. A cold wave occurs when the temperature drop within 24 h exceeds 8 °C (or the temperature drop within 48 h exceeds 10 °C, and the temperature drop within 72 h exceeds 12 °C), and the daily minimum temperature falls to 4 °C or below. Among them, a severe cold wave occurs when the temperature drop within 24 h exceeds 10 °C (or the temperature drop within 48 h exceeds 12 °C, and the temperature drop within 72 h exceeds 14 °C), and the daily minimum temperature falls below 2 °C. An extreme cold wave occurs when the temperature drop within 24 h exceeds 12 °C (or the temperature drop within 48 h exceeds 14 °C, and the temperature drop within 72 h exceeds 16 °C), and the daily minimum temperature falls to 0 °C or below. The standard for different cold waves is shown in Table 5.

According to the criteria in Table 5, this paper determines the occurrence of cold waves and classifies them based on the daily minimum temperature and the temperature drop magnitude. Considering the diversities in temperature changes, based on the temperature data from 2012 to 2019 provided by the China Meteorological Data Network (http://data.cma.cn/ accessed on 1 September 2020), this paper selects 10 typical cities to analyze the frequency of cold waves. The daily minimum temperature data of the 10 stations are processed using a self-programmed Matlab R2021a program, and the temperature data are analyzed hour by hour according to the criteria in Table 5. If the conditions for a cold wave are met, it is marked as true; otherwise, it is marked as false. When multiple words ‘true’ appear within a continuous period, it indicates that the continuous cooling process for several consecutive days satisfies the cold wave condition, indicating a long cold wave process, which should be counted as one cold wave and included in the cumulative result. The 10 cities are divided into five regions based on their geographical locations in China, as shown in Table 6. All analyses in this paper are conducted for these five regions.

### 2.4. Thermal Stress Analysis Based on Finite Element Simulation

**Figure 3:**
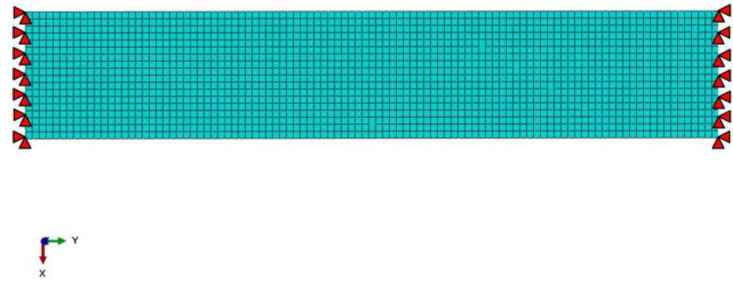
Boundary condition for FE simulation.

The thermal stress restrained specimen test (TSRST) is one of the most effective methods for studying the low-temperature cracking resistance of asphalt mixture. Therefore, this paper utilizes the finite element method to simulate the thermal stress of asphalt mixture. In Abaqus, a two-dimensional restrained specimen is established with dimensions of 40 mm × 220 mm. The model is meshed using CPE4, a four-node bilinear plane strain quadrilateral element, resulting in a total of 8800 elements. To simulate the fracture of asphalt mixture, a self-compiled program developed by MATLAB was adopted to edit the initial ‘inp file’ generated by Abaqus 6.12. In this process, the zero-thickness CZM element is inserted into the interface one by one, its element type is COH2D4. The mechanical behavior of asphalt mixture is described using a linear viscoelastic constitutive model, with the relaxation modulus and the temperature shift factor determined in Section 2.2 as input parameters. Both ends of the specimen model are completely restrained (U_1_ = U_2_ = U_3_ = UR_1_ = UR_2_ = UR_3_ = 0), ensuring that there is no relative displacement change at the ends of the specimen. The boundary condition is shown in Figure 3. The thermal and mechanical properties of the asphalt mixture are listed in Table 7.

**Figure 4:**
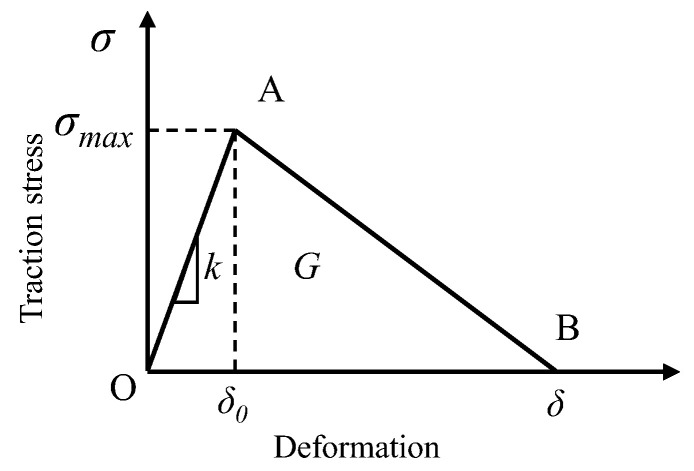
Tractor-separation curve of CZM.

Cohesive zone model (CZM) has been widely used to investigate the interface debonding process in material fracture evaluation. Therefore, the bilinear cohesive zone model was selected to describe the constitutive relation of bonding interface. Tractor-separation law is shown in Figure 4.

When the traction stress satisfies the following relationship, the cohesive element begins to be damaged.

(5) $${\left\{\frac{\langle T_{n}\rangle }{T_{n}^{0}}\right\}}^{2}+{\left\{\frac{T_{s}}{T_{s}^{0}}\right\}}^{2}=1$$

where < > is Macaulay brackets, which means compression stress will not cause damage to cohesive element. $T_{n}^{0}$ and $T_{s}^{0}$ are traction stresses in normal direction and shear direction, respectively. $T_{n}$ and $T_{s}$ are pure normal traction and pure shear traction at the beginning of damage. Once the failure starts, the material goes into a softening state. This process is quantified by defining a damage variable *D*, as shown in Equation (6).

(6) $$D=\frac{\delta_{f}(\delta_{\max}-\delta_{0})}{\delta_{\max}(\delta_{f}-\delta_{0})}$$

where $\delta_{0}$, $\delta_{\max}$, and $\delta_{f}$ are the effective displacement at the beginning of damage, the maximum effective displacement obtained during loading, and the effective displacement at the point of complete failure, respectively. In this paper, the power law failure criterion based on energy is used to describe the fracture evolution in mixed mode:

(7) $${\left\{\frac{G_{n}}{G_{n}^{c}}\right\}}^{2}+{\left\{\frac{G_{t}}{G_{t}^{c}}\right\}}^{2}=1$$

where $G_{n}^{c}$ and $G_{t}^{c}$ are the fracture energy in normal and shear directions, respectively. $G_{n}$ and $G_{t}$ are the dissipated energies generated in the normal and shear direction during the loading procedure. Gu et al. [41] studied the low-temperature fracture performances of asphalt mixtures prepared with AH-70# and AH-90# asphalt, pointing out that the difference in fracture strength for both mixtures is only 0.2 MPa, accounting for no more than 5% of the ultimate strength. Therefore, the same bonding parameters are selected for AC-13-70 and AC-13-90 mixtures in this work. The parameters of CZM model used in this paper are shown in Table 8. 

### 2.5. Prediction Method of Thermal Fatigue Life

Fatigue failure is one of the major damage forms to asphalt pavement. There are many theories for predicting the fatigue life of asphalt layer caused by traffic loads, but thermal fatigue failure is an important inducement for surface cracking. Temperature changes lead to thermal stress, and the thermal fatigue life of asphalt mixture under the action of cold waves can be predicted using the thermal fatigue equation.

Xie et al. [42] and Lv et al. [27] pointed out that in traditional fatigue equations, the tensile stress obtained when N_f_ = 1 is considered the ultimate tensile stress, which is believed to be a constant for asphalt mixture. However, this does not align with the viscoelastic nature of asphalt mixture. As the temperature changes, the ultimate tensile stress of asphalt mixtures also varies accordingly. Therefore, the ultimate tensile stress varying with temperature is selected as the cracking index in this paper, and the temperature fatigue equation under low temperatures is chosen to predict the thermal fatigue life. The temperature fatigue equations for asphalt mixtures under low temperatures are shown in Equations (8) and (9).

(8) $$\mathrm{Regions}\;1\;\mathrm{and}\;4\;[N_{f}]={\left(\frac{S_{v}}{\sigma }\right)}^{3.114}$$

(9) $$\mathrm{Regions}\;2\;,\;3\;,\;\mathrm{and}\;5\;[N_{f}]={\left(\frac{S_{v}}{\sigma }\right)}^{2.959}$$

where $\left[N_{f}\right]$ is the thermal fatigue life of asphalt mixture; $S_{v}$ represents the ultimate tensile stress of asphalt mixtures; and σ is the maximum thermal stress caused by cold wave.

## 3. Results and Discussion

### 3.1. Distributing Characteristics of Cool Wave

**Figure 5:**
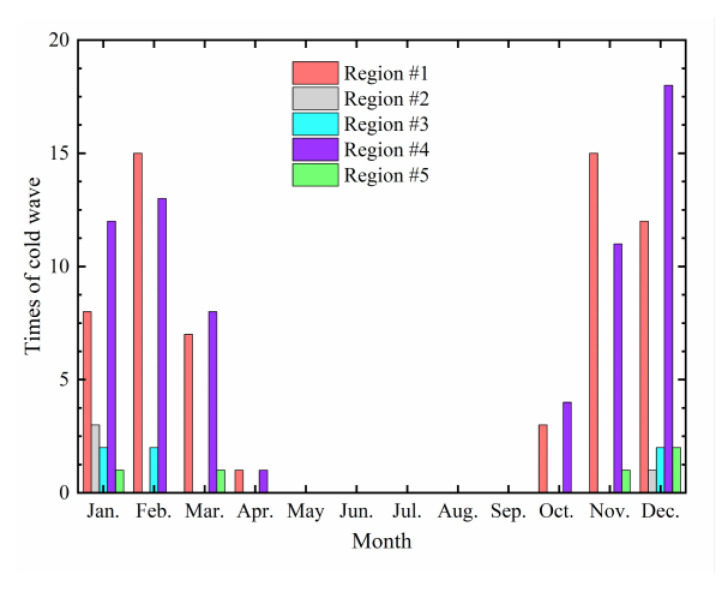
Instances of cold waves in different months from 2012 to 2019.

Using the temperature data of some meteorological stations from 2012 to 2019, statistical analysis on cold waves was conducted, and the distribution of cold waves in different months is shown in Figure 5.

As shown in Figure 5, the occurrence of cold waves in various regions is concentrated in January to March and October to December, which are roughly the winter months in the northern hemisphere. Only a few cold waves occur in spring, and none occur in summer. Regions #1 and #4 experience many instances of cold waves, corresponding to North China and Northeast China, respectively. According to the distribution trend, the months with the highest frequency of cold waves in region #1 are November and February, with similar occurrences in both months. For region #4, the month with the most cases of cold waves is December, followed by February. This indicates that the frequency and timing of cold waves vary across different regions. Based on their geographical locations, Northeast China has a higher latitude than North China, suggesting that the higher the latitude, the greater the probability of cold waves occurring in December. On the other hand, cold waves in regions #2, #3, and # 5 mainly occur from November of the previous year to February of the next year, with a maximum of three occurrences in January. This further demonstrates that the timing of cold waves varies significantly across regions, and there are also obvious differences in the frequency of cold waves (e.g., in December, region #4 experienced 18 cold waves from 2012 to 2019, while region #2 only experienced one). This implies that the change in atmospheric temperature would impose a notable impact on asphalt pavement, and this impact on the thermal fatigue life of asphalt pavement deserves further attention.

Based on the criteria of cold waves, the cold waves with the largest temperature drop were selected for statistics. The initial temperature, temperature drop, and cooling rate of different cold waves are listed in Table 9. 

As can be seen from Table 9, the temperature drops vary between different regions. According to the extreme temperature drop within 24 h and 48 h, regions #1 and #4 (North China and Northeast China) experience larger temperature drops, while the extreme temperature drops in regions #3 and #5 are higher than that of region #2. Region #2 experiences the smallest temperature drop. In terms of the mean values of temperature drop within 24 h and 48 h, region #4 still has the largest temperature drop among all regions. The mean values of region #1 are close to those of region #3. This demonstrates that there are both differences and similarities in the temperature drop among different regions. Additionally, no 72 h cold waves occurred in regions #3 and #2 from 2012 to 2019, indicating a lower probability of prolonged and continuous temperature drops in these regions. In terms of the temperature drop within 72 h, region #4 has the highest value, while the temperature drops in regions #1 and #5 are very close. Overall, region #4 has the highest temperature drop, with a value of 18.8 °C. Region #1 has a maximum temperature drop of 17 °C, region #2 has 11 °C, region #3 has 12.9 °C, and region #5 has 16.1 °C. It can be inferred that the impact of temperature drop on asphalt pavement is different from region to region. The largest impacts may be observed in regions #4, #1, and #5, which are mainly located in northern China.

**Figure 6:**
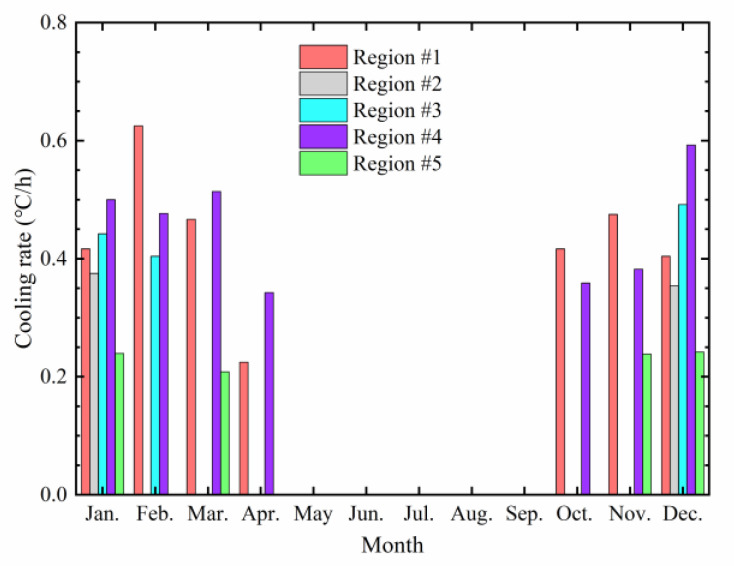
Distribution of the maximum cooling rate.

The maximum cooling rate corresponding to cold waves In each month was taken as the representative value of the cooling rate. The distribution of the cooling rate is shown in Figure 6.

As can be seen from Figure 6, the maximum cooling rates vary among different regions. The maximum cooling rates in regions #1 to #5 range from 0.2 to 0.63 °C/h. The rates in region #4 are all above 0.3 °C/h, with a maximum of 0.59 °C/h, while the maximum value of the cooling rate in region #1 reaches 0.63 °C/h, the highest among all the five regions, occurring in February. The maximum cooling rate in region #2 remains stable at around 0.39 °C/h, while the maximum cooling rate in region #3 is between 0.4 and 0.49 °C/h, and the cooling rate in region #5 ranges from 0.21 to 0.24 °C/h. Therefore, the cooling rates in regions #2, #3, and #5 are relatively constant, while the cooling rates in regions #1 and #4 vary significantly, which may lead to premature thermal fatigue failure of asphalt pavement. 

### 3.2. Simulation and Verification of Thermal Stress

**Figure 7:**
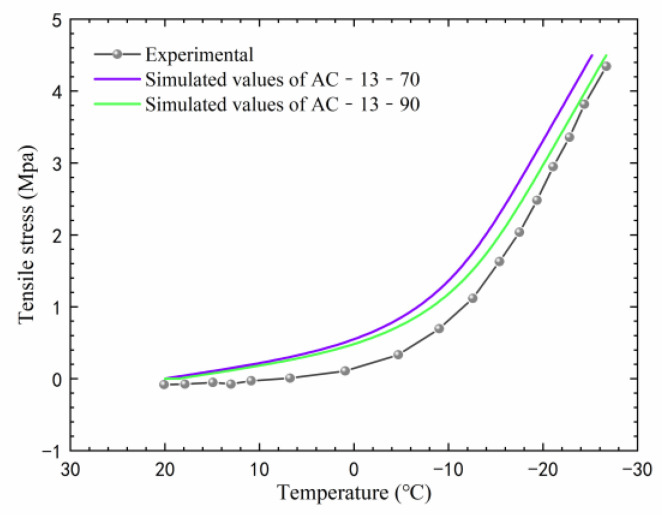
Comparison between experimental and simulated thermal stresses.

The finite element method was used to simulate the thermal stress, with an initial temperature of 20 °C and a temperature drop rate of 10 °C/h in the simulation. In this paper, a comparison was made between the simulated and the measured thermal stress reported by Zhang [43], as shown in Figure 7. 

As can be seen from Figure 7, the thermal stress simulated using the finite element method is higher than the measured one. Although the curves do not completely overlap, their trends of variation with the decrease in temperature are consistent. The final failure stress is essentially consistent with the measured value. This indicates that the thermal stress of the asphalt mixture can be predicted by the finite element method. Figure 7 also shows that the thermal stress increases as the temperature decreases. After the temperature drops below −10 °C, the growth rate of thermal stress begins to accelerate significantly. This means that when the temperature is above −10 °C, asphalt concrete has good relaxation performance, which reduces the thermal stress. However, as the temperature decreases, the asphalt binder gradually becomes harder, reducing its ability to relax thermal stress, which results in a gradual accumulation of thermal stress. Wang et al. [10] indicated that when the temperature drops below −18.6 °C, the thermal stress increases linearly. This is consistent with the findings of this study, where at this point, the thermal stress cannot be relaxed at all, and it increases linearly.

### 3.3. Comparison of Thermal Stress in Different Regions

**Figure 8:**
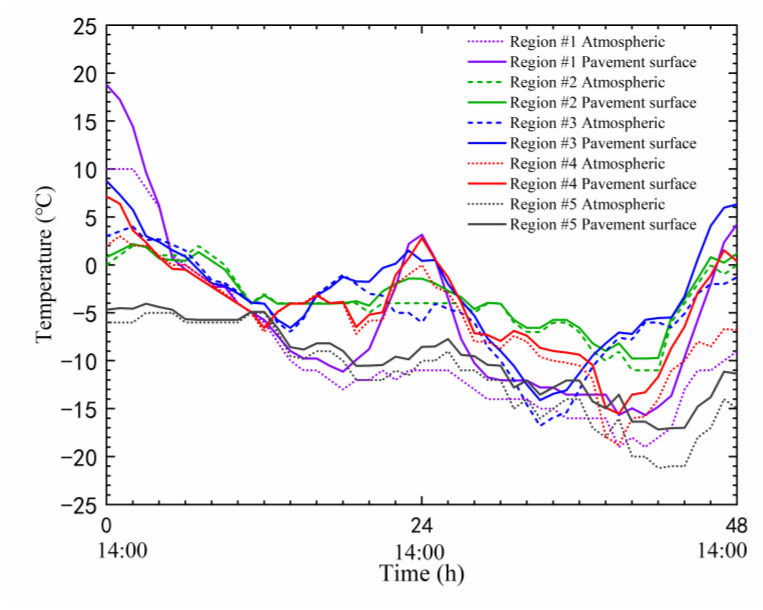
Comparison of road surface temperature and atmospheric temperature.

Qin et al. [44] pointed out that there was a quantitative relationship between road surface temperature and atmospheric temperature, as shown in Equation (10). This equation can be used to estimate the road surface temperature based on atmospheric temperature.

(10) $$T_{0}=64.9e^{0.014T_{a1}}+e^{1.16Q_{4}}-66.4$$

where *T*_0_ represents the road surface temperature; *T*_*a*1_ represents the atmospheric temperature of the previous 1 h; and *Q*_4_ is the cumulative solar radiation intensity of the previous 4 h (kW/m^2^). In this paper, this relationship is used to determine the road surface temperature based on atmospheric temperature, and the road surface temperature is used for the thermal boundary in FE simulation. The variations of road surface temperature for the typical 48 h cold waves are shown in Figure 8.

**Figure 9:**
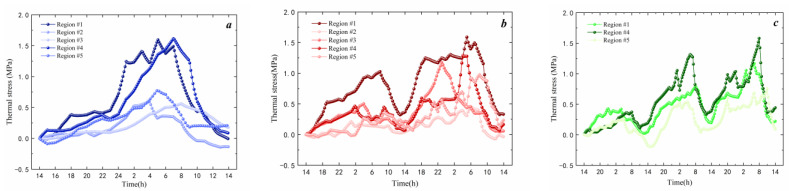
Thermal stress of asphalt mixture: (**a**) 24 h extreme cold waves; (**b**) 48 h extreme cold waves; (**c**) 72 h extreme cold waves.

As shown in Figure 8, the road surface temperature is not consistent with the atmospheric temperature in all regions. In region #1, the road surface temperature gradually rises after 8:00 a.m., and the highest difference between the road surface temperature and the air temperature is 14 °C. After 18:00, the trend of road surface temperature gradually becomes consistent with that of the air temperature, but the road surface temperature is still about 2.5 °C higher than the air temperature. Although the changes in road surface temperature in the other four regions are similar to that of region #1, the differences between road surface temperature and air temperature are not as large as in region #1. For example, in region #3, the highest difference between road surface temperature and air temperature is only 6.4 °C. This is because the weather conditions in different regions affect the cloud coverage and solar radiation, resulting in road surface temperature approaching atmospheric temperature. However, in northern China, cold waves are often accompanied by windy weather, and most of the time, after the temperature drop, it is sunny. At this time, the road surface is exposed to good solar radiation, ultimately leading to a significant increase in road surface temperature. Overall, road surface temperature is not consistent with atmospheric temperature, and when evaluating the thermal stress, it is necessary to adopt the road surface temperature as a thermal boundary for simulation. Xu et al. [45] pointed out that low-temperature cyclic action will lead to an increase in the micro-defects of asphalt mastic, and the bee structures of asphalt mastic are replaced by irregular valley formations. After the cycling action, the relaxation performance of the asphalt mastic decreases, and the bonding performance at the asphalt–aggregate interface deteriorates. Thermal stresses under the influence of extreme cold waves in each region are shown in Figure 9.

**Figure 10:**
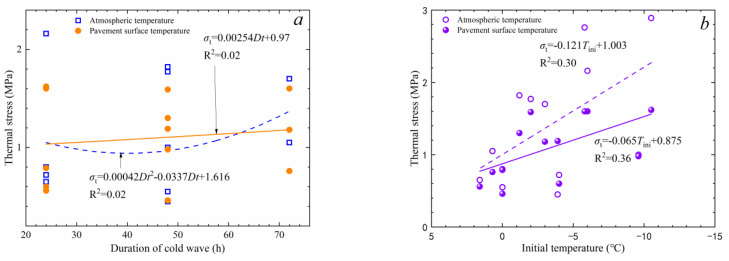

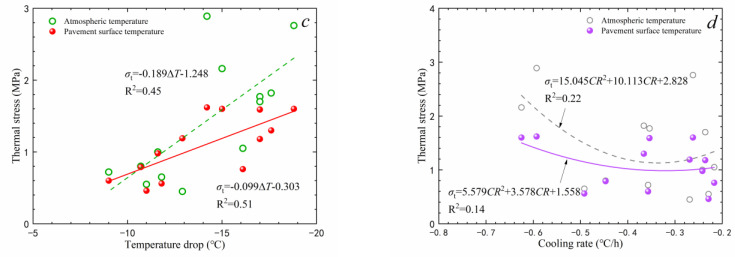
Correlation between thermal stress and cold wave parameters: (**a**) duration of cold wave; (**b**) initial temperature; (**c**) temperature drop; (**d**) cooling rate.

As can be seen from Figure 9a, the peak thermal stress during a 24 h extreme cold wave varies among different regions. Region #4 has the highest thermal stress, reaching 1.63 MPa, followed by region #1 with a maximum thermal stress of 1.6 MPa. The maximum thermal stress in the other three regions is only 0.8 MPa, approximately half of the value of regions #1 and #4. As shown in Figure 10b, region #1 has the largest accumulated thermal stress due to temperature changes, reaching 1.77 MPa. Regions #4 and #3 follow with maximum accumulated stresses of 1.3 MPa and 1.2 MPa, respectively. For the 48 h cold wave, region #2 has the smallest accumulated thermal stress, with a maximum value of 0.46 MPa. According to Figure 10c, the 72 h cold wave is only distributed in regions #1, #4, and #5. Among them, region #4 has the highest peak thermal stress, reaching 1.6 MPa, followed by region #1 with 1.18 MPa. Region #5 has a small accumulated thermal stress, only 0.76 MPa. Comparing the three conditions in Figure 10, it can be observed that thermal stress mainly peaks between 2 am and 8 am. For 48 h and 72 h cold waves, the peak thermal stress during the second and third 24 h periods are higher than those in the first 24 h period, indicating a significant accumulation of thermal stress during the cooling process. The temperature of the asphalt mixture gradually decreases as the duration of low temperature extends. When the temperature falls below the glass transition temperature of asphalt binder, the molecular chains of asphalt move slower, and the relaxation ability continues to decrease. This causes a decrease in the relaxation and an increase in thermal stress.

To determine the impact of thermal boundaries on the thermal stress of the asphalt mixture, thermal stress was simulated under atmospheric and road surface temperature conditions. The peak thermal stresses are presented in Table 10.

As listed in Table 10, the peak thermal stress using atmospheric temperature as the thermal boundary is mostly higher than those obtained using road surface temperature, with a maximum difference of 1.27 MPa (region #4, 24 h cold wave). This indicates that using atmospheric temperature as the thermal boundary for thermal analysis may overestimate the thermal stress. Comparing the occurrence moments of peak thermal stress, it can be observed that although thermal boundary conditions change the occurrence moments of peak thermal stress, the deviation of moments is no more than 2 h. To determine the influence of different factors on peak thermal stress, regression analysis was performed on the data in Table 9 and Table 10, and the results are shown in Figure 10.

As can be seen from Figure 10a, there is no correlation between the duration of the cold wave and thermal stress, indicating that the duration of the cold wave does not have a significant impact on the peak thermal stress. Figure 10b,c show that there is a moderate correlation between thermal stress and both the initial temperature and the cooling rate. The lower the initial temperature and the greater the cooling rate, the higher the thermal stress generated later. Figure 10d indicates that there is a weak correlation between the peak thermal stress and the cooling rate. Higher cooling rates lead to higher peak thermal stress. When the cooling rate is lower than −0.5 °C/h, the peak thermal stress increases rapidly. It can be inferred that the magnitude of thermal stress in asphalt pavements is primarily influenced by the temperature drop and the road surface temperature at the onset of the cold wave.

### 3.4. Prediction of Thermal Fatigue Life 

Based on the cold wave grades classified in Table 3, a statistical analysis was conducted on the frequency of cold waves in the selected region from 2012 to 2019. The results are summarized in Table 11.

As can be seen from Table 11, region #1 experiences the most instances of cold waves, followed by region #4. However, the total number of severe and extreme cold waves in region #4 is higher than that in region #1. Over the eight-year period studied, no extreme cold waves happened in regions #2 and #3. Region #5 experienced one extreme cold wave. These cold waves will generate thermal stress within asphalt pavement. However, as listed in Table 10, the peak thermal stress did not exceed its tensile strength. Therefore, the primary damage of the asphalt mixture under the influence of cold waves is likely to be caused by thermal fatigue damage. Consequently, the thermal fatigue performance analysis was conducted and the predicted thermal fatigue lives are presented in Table 12.

As can be seen from the results in Table 12, the thermal fatigue life of the asphalt mixture in region # 1 and region # 4 is the shortest, with only 18 cycles, followed by the mixture of region #3 and region #5. The asphalt mixture used in region #2 has the longest thermal fatigue life. Based on the graded principle of the general cold wave, the lowest road surface temperature is higher than 4 °C when the general cold wave occurs. The mixture has good relaxation ability at this moment, and the generated thermal stress does not exceed 0.3 MPa. Based on the predicted method of Equations (8) and (9) using 0.3 MPa, it can be determined that the fatigue life under the influence of general cold waves ranges from 909 to 1300 cycles. This means that the degree of thermal fatigue damage caused by general cold waves in the service stage is very small. Therefore, pavement thermal fatigue damage is mainly caused by severe and extreme cold waves. 

Based on cold wave data from 2012 to 2019, the total number of cold waves that the asphalt pavement may encounter within a 15-year service life is estimated and listed in Table 13. Furthermore, based on the principle of extreme working conditions, the thermal fatigue stress thresholds for the asphalt mixture in five regions are determined using Equations (8) and (9).

As can be seen from Table 13, the thermal fatigue stress thresholds vary among different regions. Region #4 has the highest thermal stress threshold, while region #2 has the lowest. The difference between the thermal fatigue stress thresholds of region #1 and region #4 is 0.04 MPa, and the thermal stress threshold of region #4 is approximately twice that of region #3. This indicates that there are significant differences in thermal fatigue performance among regions at different latitudes. As for the asphalt mixture, if the thermal stress does not exceed 0.77 MPa in regions #1 and #4, there should be no thermal fatigue damage to the asphalt layer during the service life in theory. However, to meet the requirements of thermal fatigue crack resistance within the designed lifespan (15 years for China), it is necessary to control the thermal stress in the asphalt layer. In other words, as long as the thermal stress during the use does not exceed the above threshold, it can be ensured that no thermal fatigue damage occurs in these regions within the designed lifespan of 15 years. Nevertheless, due to the influence of thermal-oxidative aging and moisture, thermal fatigue cracking in the asphalt layer would happen earlier. This requires further improvement to the prediction method in the future. 

## 4. Conclusions

This work uses atmospheric temperature data from five typical regions in China from 2012 to 2019 to analyze the frequency and moment of cold waves in different regions. Based on typical cold waves, road surface temperatures are calculated and used to analyze the thermal stress of the asphalt mixture. The thermal fatigue life of the asphalt mixture in different regions is then predicted. The following conclusions can be drawn.

There are significant differences in the frequency of cold waves in the selected five regions. Among them, the temperature drop in North China and Northeast China is the largest, with the highest frequency of severe and extreme cold waves. Cold waves mainly occur from November of one year to March of the next year, with the cooling rate ranging from 0.2 °C/h to 0.63 °C/h.

Severe and extreme cold waves result in higher thermal stress, with a clear accumulation of thermal stress during the cooling process. The peak thermal stress in different regions is not the same, and it does not exceed the ultimate tensile strength of the asphalt mixture. The maximum thermal stress in Northeast and North China can reach 1.6 MPa, while it is approximately 0.6 MPa in Central China, 1.19 MPa in East China, and 0.98 MPa in Northwest China.

Severe and extreme cold waves are the main causes of thermal fatigue damage in asphalt mixtures. There are significant differences in the thermal fatigue lives of asphalt mixtures in different regions. The thermal fatigue lives of asphalt mixtures in Northeast and North China are short, while the thermal fatigue life of asphalt mixtures in Central China, East China, and Northwest China is long. Within the designed service life, asphalt mixtures in these regions will not experience thermal fatigue damage. It is recommended that the thermal fatigue stress threshold in Northeast and North China be set at 0.77 MPa, while in other regions, it should be 0.39 MPa.

This work clarifies the distribution of cold waves, determines the impact of cold waves on the thermal fatigue life of the asphalt mixture, and provides a technical approach for the design of thermal fatigue resistance of asphalt mixtures. In the future, the influence of asphalt with greater penetration, aggregate types, gradations, and binder content should be investigated, and more variables such as pavement structures in the FE simulation should also be conducted. 

## Figures and Tables

**Table 1:** Properties of AH-70# and AH-90# asphalt.

Items	AH-70#	AH-90#	Test Methods
Penetration (25 °C, 0.1 mm)	71	87	ASTM D5 [30]
Softening point (°C)	47.5	45.5	ASTM D36 [31]
Ductility (15 °C, cm)	>100	>100	ASTM D113 [32]
Flashing point (°C)	285	281	ASTM D92 [33]
Mass loss after TFOT, 163 °C, 5 h (%)	0.15	0.38	ASTM D6 [34]
Penetration ratio after TFOT (%)	78.8	72	ASTM D5 [30]
Ductility after TFOT (cm)	6.5	7.4	ASTM D113 [32]
G* @ 58 °C (kPa)	6.78	5.99	ASTM D7175 [35]
δ @ 58 °C (◦)	81.7	80.5	/
G*/sin δ @ 58 °C (kPa)	6.86	6.07	/

**Table 2:** Properties of aggregates.

Sieve Size (mm)	13.2	9.5	4.75	2.36	1.18	0.6	0.3	0.15	0.075
Apparent gravity (g/cm^3^)	2.723	2.681	2.679	2.694	2.708	2.713	2.713	2.712	2.712
Moisture uptake ratio (%)	0.57	0.63	0.52	-	-	-	-	-	-

**Table 3:** Viscoelastic parameters of asphalt mixture@20 °C.

Mixture Type	*i*	*E_i_*	$\tau_{i}$
AC-13-70	∞	41.99	/
	1	1214	0.2
	2	6.08 × 10^−7^	2
	3	25.08	20
	4	13.37	200
	5	0.02903	2000
AC-13-90	∞	1.078 × 10^−9^	/
	1	309.6	0.2
	2	5016	2
	3	265.7	20
	4	3.75 × 10^−10^	200
	5	9.09 × 10^−10^	2000

**Table 4:** Temperature shift factor parameters of asphalt mixture.

Reference Temperature T_0_ (°C)	C_1_	C_2_
20	5	70

**Table 5:** Standard for cold waves.

Level	Daily Minimum Temperature (°C)	Temperature Drop (°C)
Cold wave	T_D_ ≤ 4	8 ≤ ΔT_24_ < 10
		or 10 ≤ ΔT_48_ < 12
		or 12 ≤ ΔT_72_ < 14
Severe cold wave	T_D_ ≤ 2	10 ≤ ΔT_24_ < 12
		or 12 ≤ ΔT_48_ < 14
		or 14 ≤ ΔT_72_ < 16
Extreme cold wave	T_D_ ≤ 0	ΔT_24_ ≥ 12
		or ΔT_48_ ≥ 14
		or ΔT_72_ ≥ 16

**Table 6:** Classification of typical regions.

Region No.	Region Name	Typical Cities	Mixture Type
1#	North China	Beijing, Hohhot	AC-13-90
2#	Central China	Wuhan, Zhengzhou	AC-13-70
3#	East China	Shanghai, Jinan	AC-13-70
4#	Northeast China	Shenyang, Harbin	AC-13-90
5#	Northwest China	Xining, Urumqi	AC-13-70

**Table 7:** Thermal and mechanical properties of asphalt mixture.

Mixture Type	Thermal Shrinkage Coefficient (×10^−6^)	Elastic Modulus (MPa)	Poisson’s Ratio
AC-13-70	37	360	0.3
AC-13-90	37	310	0.3

**Table 8:** Parameters of CZM model.

Mixture Type	Temperature (°C)	Tensile Strength (MPa)	Fracture Energy (J/m^2^)
AC-13-70 & AC-13-90	−30	4.54	314
	−10	4.06	314
	0	3.32	760
	10	2.58	1631

**Table 9:** Characteristics of cold waves with the largest temperature drop.

Region No.	Duration (h)	Initial Temperature (°C)	Extreme Temperature Drop (°C)	Cooling Rate (°C/h)	Mean Value of Temperature Drop (°C)
#1	24	−6	−15	−0.625	−9.2
	48	−2	−17	−0.354	−11.4
	72	−3	−17	−0.236	−13.7
#2	24	−4	−9	−0.357	−8.8
	48	0	−11	−0.229	−10.5
	72	None	None	None	None
#3	24	1.6	−11.8	−0.492	−9.3
	48	−3.9	−12.9	−0.269	−11.2
	72	None	None	None	None
#4	24	−10.5	−14.2	−0.593	−10
	48	−1.2	−17.6	−0.366	−12.3
	72	−5.8	−18.8	−0.262	−14.3
#5	24	0	−10.7	−0.447	−8
	48	−9.6	−11.6	−0.242	−10.9
	72	0.7	−16.1	−0.217	−13.9

**Table 10:** Peak thermal stresses and occurrence moment under different boundaries.

Thermal Boundary	Duration	Property	Region #1	Region #2	Region #3	Region #4	Region #5
Atmospheric temperature	24 h	Thermal stress (MPa)	2.16	0.72	0.65	2.89	0.8
		Occurrence moment	5 a.m.	4 a.m.	10 a.m.	7 a.m.	5 a.m.
	48 h	Thermal stress (MPa)	1.77	0.55	0.45	1.82	1.0
		Occurrence moment	3 a.m.	6 a.m.	11 p.m.	5 a.m.	8 a.m.
	72 h	Thermal stress (MPa)	1.7	/	/	2.76	1.05
		Occurrence moment	5 a.m.	/	/	7 a.m.	10 a.m.
Road surface temperature	24 h	Thermal stress (MPa)	1.60	0.60	0.56	1.62	0.79
		Occurrence moment	4 a.m.	4 a.m.	8 a.m.	6 a.m.	5 a.m.
	48 h	Thermal stress (MPa)	1.59	0.46	1.19	1.30	0.98
		Occurrence moment	5 a.m.	4 a.m.	11 p.m.	4 a.m.	6 a.m.
	72 h	Thermal stress (MPa)	1.18	/	/	1.60	0.76
		Occurrence moment	6 a.m.	/	/	7 a.m.	10 a.m.

**Table 11:** Statistics of cold waves in different regions from 2012 to 2019.

Region	#1	#2	#3	#4	#5
Total number	50	4	5	39	4
Number of cold waves	34	4	3	19	3
Number of severe cold waves	7	0	2	12	0
Number of extreme cold waves	9	0	0	8	1

**Table 12:** Predicted thermal fatigue lives.

Region	#1	#2	#3	#4	#5
Initial temperature (°C)	−6	−4	−3.9	−10.5	−9.6
Peak thermal stress (MPa)	1.6	0.64	1.18	1.63	1
Ultimate tensile stress *S*_v_ (MPa)	4.10	3.89	3.99	4.17	4.14
[*N_f_*]	18	207	36	18	67

**Table 13:** Estimated thresholds for thermal fatigue stress.

Region	#1	#2	#3	#4	#5
Estimated cases of severe and extreme cold waves in 15 years	30	0	4	38	2
Ultimate tensile stress *S*_v_ (MPa)	4.10	3.89	3.99	4.17	4.14
Thermal fatigue stress threshold (MPa)	0.73	0.000	0.39	0.77	0.30

## Data Availability

Data are contained within the article.
